# Clinical Outcomes of Topical Bevacizumab for the Treatment of Corneal Neovascularization

**DOI:** 10.7759/cureus.59548

**Published:** 2024-05-02

**Authors:** Mohd Ihsan Jamaludin, Wan Haslina Wan Abdul Halim, Teck Chee Cheng

**Affiliations:** 1 Ophthalmology, Universiti Kebangsaan Malaysia Medical Centre, Kuala Lumpur, MYS

**Keywords:** topical steroids, non-steroidal anti-inflammatory drugs, corneal neovascularisation, bevacizumab therapy, anti-vascular endothelial growth factor

## Abstract

Background and objective

In corneal neovascularization, the peri-corneal vascular structure grows into a normally avascular cornea. This is due to an imbalance between the angiogenic and anti-angiogenic factors that sustain corneal transparency. There are various etiologies of this condition, and they can be divided into infective or non-infective causes, such as inflammation, trauma, or surgical causes. Corneal neovascularization has been shown to improve with the current treatments using steroids and anti-vascular endothelial growth factors. This study aimed to evaluate the effectiveness of topical bevacizumab as an anti-angiogenic agent in patients with corneal neovascularization.

Methods

This retrospective study included patients who suffered corneal neovascularization of various etiologies and completed six months of topical bevacizumab therapy between 2020 and 2022 at the Universiti Kebangsaan Malaysia Medical Centre.

Results

A total of 16 patients received treatment with topical bevacizumab over the three-year study period. Based on specified inclusion and exclusion criteria, 12 patients were eligible for inclusion in this study. Eight patients (66%) showed improvement in terms of either ‘clock hours’ of improvement, morphology, or regression of corneal neovascularization. All infective causes of corneal neovascularization showed improvement on completion of bevacizumab compared to other causes.

Conclusion

Topical bevacizumab can be one of the treatment choices for corneal neovascularization. As the outcome varies depending on the severity and chronicity of the condition, the attending ophthalmologist should treat each case differently. Although topical bevacizumab is more effective in mild and moderate cases, the indications for its use in chronic cases remain debatable as the results are unfavorable in such cases.

## Introduction

Corneal neovascularization is a vision-threatening condition in which the peri-corneal vascular structure grows into a normally avascular cornea [1], thus causing a disturbance in the normal anatomy and leading to corneal opacity. This is due to an imbalance between the angiogenic and anti-angiogenic factors that sustain corneal transparency [2]. It occurs in various corneal pathologies, such as congenital corneal diseases, contact lens-related hypoxia, inflammatory disorders, chemical burns, limbal stem cell deficiency, allergic conjunctivitis, ocular trauma, infectious keratitis, autoimmune diseases, and corneal graft rejection [1]. Current therapies aimed at regressing corneal vessels, including amniotic membrane transplantation, topical steroid, calcineurin inhibitor or non-steroidal anti-inflammatory drug (NSAID) application, argon laser, photodynamic therapy, fine needle diathermy, and cautery, are not uniformly effective but also do come with side effects. More recently, the use of anti-vascular endothelial growth factors (VEGFs) has become a popular treatment modality for managing corneal neovascularization alongside topical steroids.

VEGF-A overexpression has been observed in corneal neovascularization caused by hypoxia and inflammation [3]. VEGF-A is a known regulator of angiogenesis and vessel permeability. It binds to VEGFR-1 and VEGFR-2 receptors, and its expression is strictly regulated [4]. Targeting VEGF-A has recently been proven to be an effective therapy for diseases related to pathological angiogenesis in clinical trials [5,6]. Anti-VEGF drugs inhibit VEGFs, thus inhibiting new blood vessel growth by downregulating endothelial cell proliferation. Selective targeting of these angiogenic growth factors is more desirable than using steroids because of their fewer side effects and more selective action. Bevacizumab is a humanized monoclonal antibody that binds to all VEGF isoforms [7], and its use seems to be an effective and safe method for treating corneal neovascularization, either through subconjunctival injections or topical application [8]. Topical bevacizumab is used off-label in Malaysia. The Universiti Kebangsaan Malaysia Medical Centre is the only medical center in Malaysia that practices topical bevacizumab treatment for corneal neovascularization. Thus, this study aimed to evaluate the effectiveness of topical bevacizumab as an anti-angiogenic agent in patients with corneal neovascularization.

## Materials and methods

Study design

This was a retrospective study.

Inclusion and exclusion criteria

All patients receiving topical bevacizumab for corneal neovascularization for a minimum treatment period of six months between January 2020 and December 2022 were included. 

The exclusion criteria were as follows: patients on treatment for less than six months and patients who used medication for more than six months but were not on medication for more than one month during treatment.

Consent

Verbal consents were obtained from each patient.

Data collection

The universal sampling method was used for all cases receiving topical bevacizumab from the pharmacy records. Information such as demographic data and indications for the use of topical bevacizumab were collected from patient records. The topical bevacizumab concentration used was 0.5%, and it was prepared by the pharmacist. Medications were stored inside the refrigerator between 2 and 8 degrees Celsius. Each patient was asked to instill the medication every four hours for 2 to 4 weeks before tapering to every four hours. In the third month, medication was tapered to every six hours and maintained until the completion of six months. Anterior segment photographs were traced from the anterior segment camera to compare corneal neovascularization before and six months after treatment. The results were reported based on an assessment of corneal ‘clock hours’ of improvement defined by the regressed corneal neovascularization based on the anatomical clock hours of the cornea, vessel morphology defined by obvious shortening and reduction of vessel engorgement seen in comparison with the pre-treatment anterior segment photo, and lastly, the presence of vessel regression defined by partial or complete regression of corneal neovascularization on the anterior segment photo after completion of treatment for six months.

Statistical analysis

The results were summarized into frequencies, represented as simple percentages. No inferential statistics were performed.

## Results

In all, topical bevacizumab eye drops were used in 16 patients for corneal neovascularization between 2020 and 2022. Of the 16 patients, only 12 had completed the treatment with topical bevacizumab for six months. For the remaining patients, either they defaulted at their subsequent follow-up, or the medication was discontinued sooner than six months because of disease progression requiring other treatment modalities.

Of the 12 patients who completed six months of topical bevacizumab treatment, three patients (25%) underwent penetrating keratoplasty, two patients (16.7%) had vernal keratoconjunctivitis (VKC), and the others had various types of infective keratitis and inflammatory disorders, such as Stevens-Johnson syndrome or toxic epidermal necrolysis (TEN).

A total of eight patients (66%) showed improvement or reduction in the degree of corneal neovascularization, including one patient who showed almost complete regression. Of the eight patients showing improvement, six patients (50%) showed improvement in clock hours, five patients (41.7%) showed some degree of vessel regression, and all eight patients (100%) showed improvement or changes in vessel morphology. Table 1 summarizes the diagnosis and outcomes based on the improvement of vessels in corneal ‘clock hours’, the morphology of corneal neovascularization, and the regression of vessels.

**Table 1:** Diagnosis and outcome of topical bevacizumab treatment * Topical steroids and calcineurin inhibitors that were started. Table 1 shows the diagnosis and outcomes based on the improvement of vessels in corneal ‘clock hours’, the morphology of corneal neovascularization, and the regression of vessels.

Demographic details	Diagnosis	Improvement	‘Clock hours’ of improvement	Morphology of vessels	Regression of vessels	Other medication*
68 y/o male	Perforated corneal ulcer, post-penetrating keratoplasty	Yes	7 ‘clock hours’	Less engorged, shorter	Yes	Ciclosporin 0.5% Loteprednol 0.5%
14 y/o female	Herpetic corneal ulcer	Yes	Nil	Less engorged	No	Ciclosporin 0.5%
28 y/o male	Ocular surface disease with limbal stem cell deficiency secondary to Stevens-Johnson syndrome	Yes	Nil	Less engorged, shorter	Yes	Ciclosporin 0.5% Dexamethasone 0.1%
15 y/o male	Vernal keratoconjunctivitis	Yes	2 ‘clock hours’	Complete regression	Yes	Ciclosporin 1% Dexamethasone 0.1%
49 y/o female	Ocular surface disease secondary to Stevens-Johnson syndrome	Yes	1 ‘clock hour’	Less engorged, shorter	Yes	Ciclosporin 1% Dexamethasone 0.1%
68 y/o female	Ocular cicatricial pemphigoid disease with severe dry eyes	No	Nil	No changes	No	Ciclosporin 1% Dexamethasone 0.1%
19 y/o male	Vernal keratoconjunctivitis with shield ulcer	No	Nil	More elongated	No	Ciclosporin 1%
22 y/o male	Ocular surface disease secondary to systemic inflammatory disease	No	Nil	Increase vascularization	No	Ciclosporin 0.5%
78 y/o male	Multiple penetrating keratoplasty with recurrent graft failure secondary to cytomegalovirus endothelitis	No	Nil	No changes	No	Ciclosporin 0.5% Dexamethasone 0.1%
34 y/o male	Severe ocular surface disease secondary to toxic epidermal necrolysis	Yes	1 ‘clock hours’	Less engorged	No	Ciclosporin 0.5%
30 y/o male	Post-penetrating keratoplasty	Yes	4 ‘clock hours’	Less engorged	No	Ciclosporin 0.5% Pred forte 1%
28 y/o female	Acanthamoeba keratitis	Yes	2 ‘clock hours’	Less engorged, shorter	Yes	Dexamethasone 0.1%

All six cases of infection and drug reactions showed 100% improvement. Only one patient (50%) with VKC and one patient (33%) with post-penetrating keratoplasty showed improvement. Patients with other causes of corneal neovascularization, such as severe ocular surface disease secondary to systemic disease, did not show any improvement.

**Figure 1:**
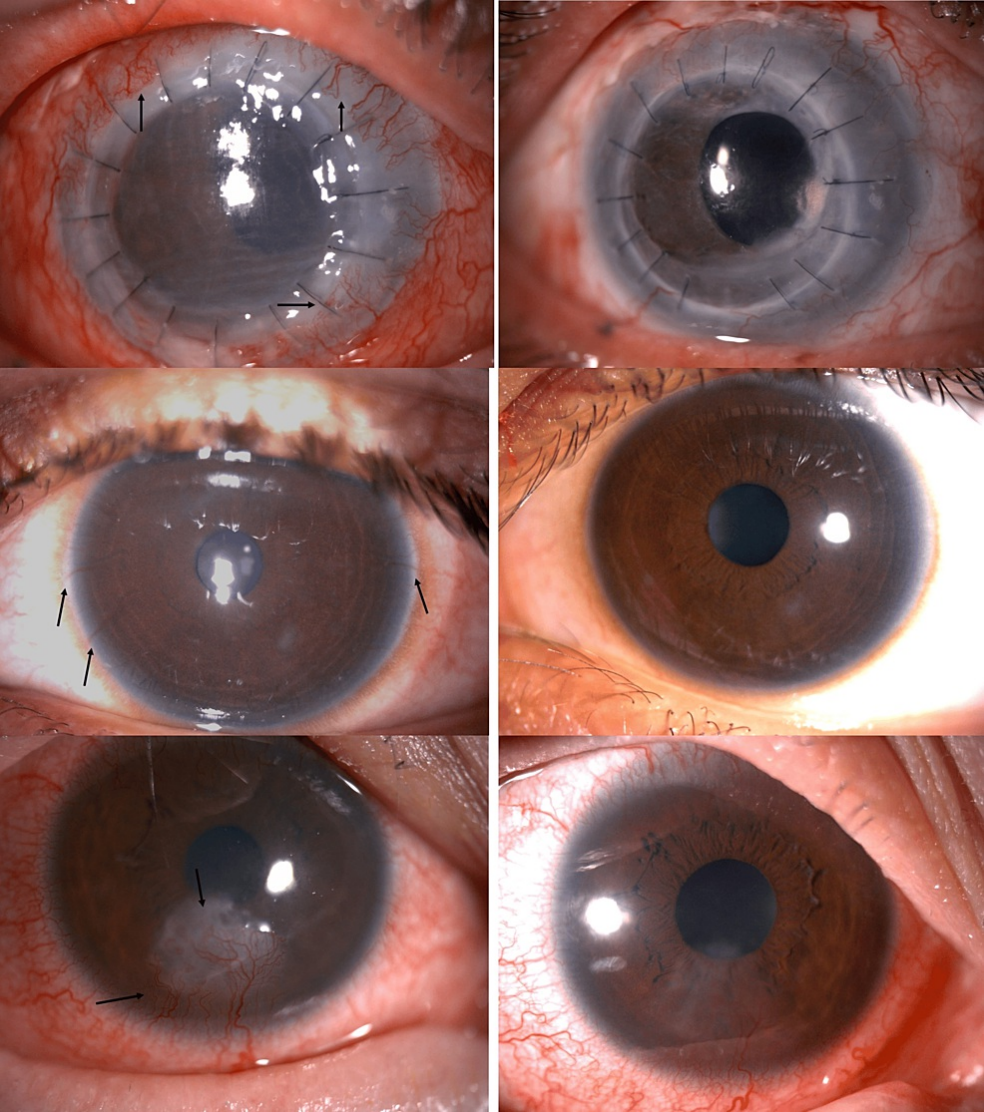
Comparison images of the pre- and post-treatment. Images on the left are pre-treatment images showing areas of corneal neovascularization (black arrows), and images on the right-side show improvement of corneal neovascularization six months post-treatment with topical bevacizumab. The corneal neovascularization either shows improvement or has completely regressed.

Figure 1 shows the improvement in corneal neovascularization in the patients comparing pre- and post-completion of six months of topical bevacizumab.

## Discussion

Steroid and anti-VEGF

Corneal neovascularization must be considered a condition that can cause further complications and halt disease recovery. The treatment can be administered either medically or surgically. Topical corticosteroids, calcineurin inhibitors, and NSAIDs are the main medical treatments. However, corticosteroids should always be used with caution in cases of infection, such as in patients with keratitis, patients who have undergone keratoplasty, and patients with glaucoma. NSAIDs help partially block severe inflammatory corneal neovascularization but delay corneal wound healing, similar to corticosteroids [9,10]. Anti-VEGF agents, such as bevacizumab, have been used as an off-label therapy for corneal neovascularization as a second-line treatment. In this observational study, the success rate of corneal neovascularization improvement was up to 67%. On comparing the findings with other studies, Koenig et al. reported a 61% reduction in the mean vascularized area and a 24% reduction in blood vessel diameter [11]. Dastjerdi et al. reported a 47.1% decrease in mean neovascular area and a 54.1% decrease in blood vessel caliber [12]. Other studies have also reported improvement following topical bevacizumab treatment and its efficacy in reducing corneal neovascularization [13].

Severity and chronicity of cases

Our study results showed that the use of bevacizumab is more effective in mild-to-moderate corneal neovascularization cases. Of the 12 patients reported here, eight showed improvement, and the remaining four showed no improvement after topical bevacizumab use for six months. The four patients had chronic, established corneal neovascularization with multiple etiologies, such as ocular cicatricial pemphigoid disease, and multiple histories of graft failure. Bevacizumab is most efficient against actively growing corneal neovascularization, and established corneal vessels do not depend on VEGF anymore. They produce fewer inflammatory factors and respond less well to the bevacizumab treatment [11]. Animal studies have shown that bevacizumab significantly inhibits corneal neovascularization in the early and mid stages but not in the late stages [14,15]. The effect of bevacizumab may also be short-lived, as the improvement is mainly noted within the first two weeks of use [16]. The use of bevacizumab is seen to be more effective in early mild-to-moderate cases compared with chronic corneal neovascularization [14].

Duration of medication

Topical bevacizumab use remains off-label, and the medication is quite expensive in Malaysia. The duration of medication use plays an important role in determining whether a patient will benefit from using the medication for a longer period. The cut-off point for our patients was six months, which is neither too short nor too long. According to Kim et al., the effect of topical bevacizumab on corneal neovascularization can be observed as early as two weeks of use and lasts for up to three months [17]. Dastjerdi et al. reported that most changes occurred within the first three weeks of topical bevacizumab treatment, and only minimal changes were noted when treatment was extended up to six months; using two drops versus four drops per day showed no significant difference in efficacy [12]. Koenig et al. reported a success rate of 61% in the reduction of corneal neovascularization with topical bevacizumab, and the medication was well tolerated up until 12 months, with a mean usage of 3.4 ± 2.9 months. It is also relatively safe and a well-tolerated option in the treatment of corneal neovascularization [11]. There is no exact duration or frequency of topical bevacizumab used, and its side effects, including corneal epithelial defects, are minimal and well-tolerated [11]. The cost-effectiveness of the medication, however, should be carefully considered.

The present retrospective study has some limitations. Most of the patients were on topical steroids or topical immunosuppressants, either concurrently with bevacizumab or before starting bevacizumab treatment. The improvement in corneal neovascularization may also result from the use of topical steroids or immunosuppressants, especially in the case of inflammation-induced angiogenesis. The indication for starting topical bevacizumab was to avoid long-term steroid-related complications, but in most cases, it acts as an adjunct therapy to it. Further, the sample size was small; thus, the results may not be sufficient for proper analysis. A larger number of patients is required, and further studies should be conducted to obtain better statistical and significant outcomes.

## Conclusions

Topical bevacizumab is indicated as a second-line medical treatment to reduce corneal neovascularization and is seen to be effective, especially in post-infective and drug reaction cases. Based on the results and in comparison with other studies, the frequency and duration of topical bevacizumab treatment should be properly determined by the attending ophthalmologist as per the indication, considering the cost-effectiveness and side effects. Although topical bevacizumab is more effective in mild and moderate cases, indications for its use in chronic cases remain debatable as the outcome of the study is unfavorable in such cases.
